# 201. Characteristics of Streptococcal Toxic Shock Syndrome Caused by Different Beta-Hemolytic Streptococci Species: A Single-Center Retrospective Study

**DOI:** 10.1093/ofid/ofad500.274

**Published:** 2023-11-27

**Authors:** Makoto Inada, Noriko Iwamoto, Hidetoshi Nomoto, Shinya Tsuzuki, Norihiko Takemoto, Noriko Fuwa, Ataru Moriya, Norio Ohmagari

**Affiliations:** National Center for Global Health and Medicine, Shinjuku-ku, Tokyo, Japan; Disease Control and Prevention Center, Tokyo, Tokyo, Japan; Disease Control and Prevention Center, National Center for Global Health and Medicine, Tokyo, Japan, Shinjuku-ku, Tokyo, Japan; National Center for Global Health and Medicine, Shinjuku-ku, Tokyo, Japan; National Center for Global Health and Medicine, Shinjuku-ku, Tokyo, Japan; National Center for Global Health and Medicine, Shinjuku-ku, Tokyo, Japan; National Center for Global Health and Medicine, Shinjuku-ku, Tokyo, Japan; National Centre for Global Health and Medicine, Shinjuku, Tokyo, Japan

## Abstract

**Background:**

Streptococcal toxic shock syndrome (STSS) is a life-threatening condition caused by beta-hemolytic streptococci (BHS) with high mortality. Initially, it is described to be associated with *Streptococcus pyogenes* and its data and evidence were constructed based on *S. pyogenes* cases. Now many studies reported that other BHS, *S. agalactiae* or *S. dysgalactiae* could also cause STSS, but the clinical characteristics of STSS caused by other BHS were poorly understood. This study evaluates the likelihood of STSS development compared to non-STSS invasive infection among different streptococcal species.

**Methods:**

We retrospectively searched for the adult medical records of invasive BHS cases in our hospital during 2002-2022. *S. pyogenes, S. agalactiae,* and *S. dysgalactiae* were categorized by the Lancefield group (GAS, GBS, and GGS, respectively). Each case was reviewed by infectious diseases specialists and classified into STSS or non-STSS groups. We conducted a multivariable analysis with bacterial species adjusted with age and diabetes mellitus, which were known risk factors. Also, GAS cases were propensity-matched (1:4) to non-GAS BHS cases.

**Results:**

We identified 43 STSS cases and 285 non-STSS cases and the median [interquartile range] age was 74.0 [63.0-85.0] and 68.0 [57.5-76.5], percentage of men was 55.1% and 46.5%, respectively. We stratified the STSS cases with bacterial species; each GAS, GBS, and GGS accounted for 17, 13, and 13 cases. The crude mortality was around 35% in all groups (Table 1). Multivariable analysis suggested that STSS was less frequent in non-GAS BHS cases with odds ratio 0.24 (95% confident interval (CI): 0.10-0.54, p< 0.001) in GBS and 0.23 (95% CI: 0.10-0.55, p< 0.001) in GGS (Table 2). After propensity score matching, *S. pyogenes* seemed to cause STSS development more likely than other BHS cases, with odds ratio 3.28 (95% CI:1.21-8.77, p=0.010).

**Table 1.**
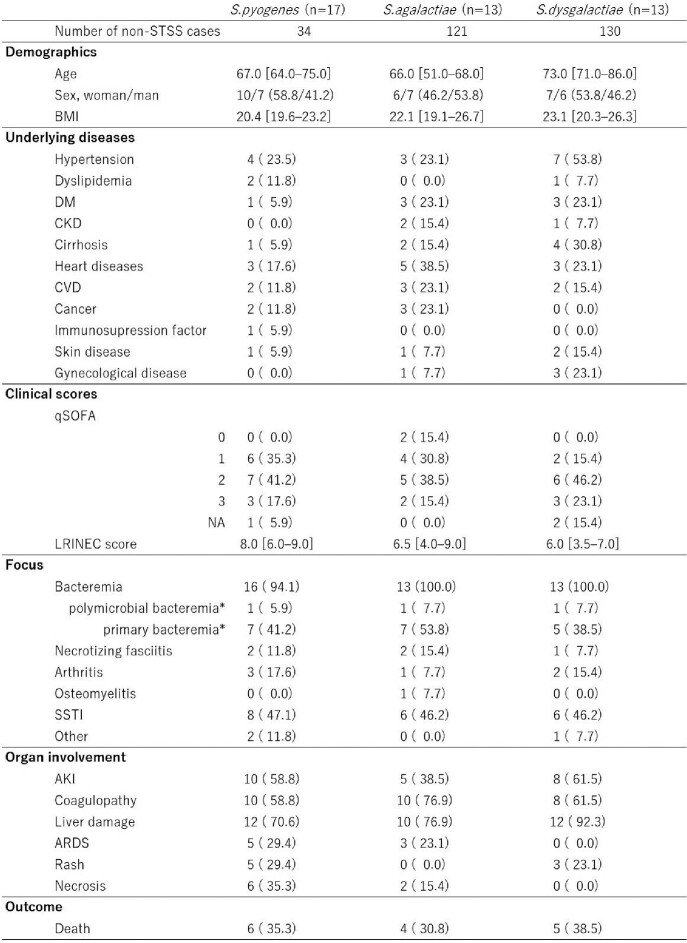
The characteristics of the STSS patients stratified by bacterial species. Data demonstrated as median [interquartile range] or number (percentage). Abbreviations: BMI, body mass index. DM, diabetes mellitus. CKD, chronic kidney disease. CVD, cerebrovascular disease. SOFA, sequential organ Failure assessment. NA, not applicable. AKI, acute kidney injury. ARDS, acute respiratory distress syndrome. * n=out of proven bacteremia cases

**Table 2.**
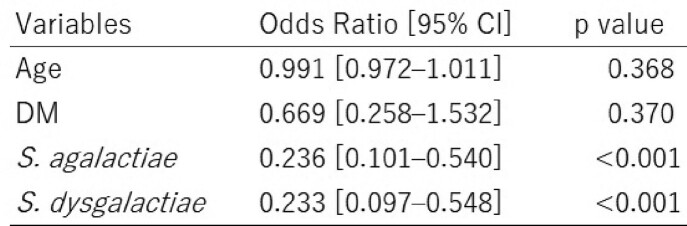
Multivariable logistic regression analysis of STSS risk factors. Abbreviations: CI, confidence interval. DM, diabetes mellitus.

**Conclusion:**

This study is the first report to describe and compare the clinical characteristics of STSS caused by different BHS, and we demonstrated that *S. pyogenes* could be more likely to cause STSS than other BHS. Further studies for assessing the similarity and differences of STSS among different bacteria would be needed for better comprehension, prevention, and treatment of STSS.

**Disclosures:**

**All Authors**: No reported disclosures

